# Living with endometriosis: a phenomenological study

**DOI:** 10.1080/17482631.2020.1822621

**Published:** 2020-09-23

**Authors:** Teresa Rea, Pierluigi Giampaolino, Silvio Simeone, Gianluca Pucciarelli, Rosaria Alvaro, Assunta Guillari

**Affiliations:** a Department of Hygiene and Public Health, A.O.U. Federico II, Naples, Italy; b Department of Biomedicine and Prevention, University of Rome Tor Vergata, Rome, Italy

**Keywords:** Endometriosis, experience, women’s health

## Abstract

**Purpose:** To explore and understand the lived experiences of women with endometriosis.

**Method:** Qualitative study using Cohen phenomenology.

**Results:** The data analysis identified four main themes and two sub-themes. The main themes are: delay in diagnosis, which includes the sub-theme of the misunderstanding of one’s state; worsening of one’s life, which includes the sub-theme of a painful life; disastrous intimate life with one’s partner; and uncertainty about being able to have one’s own children.

**Conclusions:** The themes that emerged represent the starting point for further research and for the implementation of specific educational and support strategies that improve self-care, commitment and quality of life for women with endometriosis.

## Introduction

Endometriosis is a disease where endometrial tissue forms in the ectopic area, which induces a local inflammatory response (De Nardi & Ferrrari, 2011; Dunselman et al., 2014; Kennedy et al., 2005). To date, the exact aetiology of the condition has not yet been made clear, although there are several aetiopathogenetic hypotheses. Recent evidence suggests hat genetic and epigenetic mutations play a significant role in altering the correct organogenesis processes of the reproductive system (Damewood et al., 1997; World Endometriosis Society & Foundation, 2012). Unlike the latest evidence on the aetiology of the condition, the signs and symptoms of endometriosis are well known: in some women it manifests itself with symptoms such as pain or infertility, while in others it can be asymptomatic, causing it not to be diagnosed early. The delay in diagnosis after the onset of symptoms varies from 4 to 12 years (Hadfield et al., 1996; Hudelist et al., 2012; Santos et al., 2012).

In literature, visual inspection through laparoscopy, preferably with histological confirmation, is considered the gold standard for diagnosing endometriosis (Hsu et al., 2010; Johnson et al., 2013; “Treatment of pelvic pain associated with endometriosis: a committee opinion,” 2014). However, an accurate gynaecological examination performed by experienced endometriosis specialists with high-sensitivity ultrasound could facilitate early diagnosis.

Three main types of endometriosis have been described depending on the “ectopic” site of the endometrial tissue: deep endometriosis, peritoneal endometriosis and ovarian endometrioma. Endometriosis may also be found in ‘atypical’sites (Dunselman et al., 2014; Nisolle & Donnez, 1997; SIGO, AOGOI, & AGUI, 2018). In many cases the types of lesions can coexist; however this separation is useful for diagnostic and therapeutic orientation (Giudice & Kao, 2004). Being an oestrogen-dependent pathological condition, it tends to regress post menopause. It estimates that 70 million women are affected by the disease worldwide. The prevalence of endometriosis is difficult to ascertain, but it has been estimated to be between 2% and 17% of the female population, mainly of childbearing age (Bernuit et al., 2011; Haas et al., 2012). The statistics also show that around 50% of infertile women have endometriosis (Carvalho et al., 2013; Meuleman et al., 2009). It is one of the main causes of hospital admission in developed countries (Sao Bento & Moreira, 2017) and the direct and indirect costs related to endometriosis are comparable to those of other main chronic conditions such as diabetes, Crohn’s disease and rheumatoid arthritis (Simoens et al., 2012).

There is no correlation between the category of the disease and the severity of its symptoms (Denny, 2009). Chronic and recurrent pain, dysmenorrhoea, non-menstrual pelvic pain, dyspareunia and dyschezia are the main symptoms associated with the disease; these symptoms occur in about 80% of cases. Furthermore, endometriosis can be associated with nausea, diarrhoea, sleep disturbances and fatigue (DiVasta et al., 2018; Moradi et al., 2014), affecting the quality of life of the women who suffer from it (Bourdel et al., 2015; Chauvet et al., 2017; Facchin et al., 2015; Grogan et al., 2018). Often the diagnosis of endometriosis is late, due to the variability of the lesion and the range of symptoms that can occur in addition to the common link with pelvic pain during normal menstruation (Denny & Mann, 2008).

There is currently no cure for endometriosis; treatments focus on managing symptoms. Treatments include either progestogens, oestroprogestogens or both to inhibit the growth of the disease and surgical removal of lesions by ablation or excision. These interventions have significant side effects and do not generally provide long-term relief (Dunselman et al., 2014). For these reasons, endometriosis is experienced as a chronic painful disease, often with failed diagnoses and ineffective treatments; a disease that undermines fertility, sexual experience, personal relationships and daily life (Ballweg & Endometriosis Association, 1995; Moradi et al., 2014). The quality of life of women with endometriosis is a growing concern which is increasingly expressed by both healthcare professionals and patients (Chauvet et al., 2018).

However, although several studies (Ceran et al., 2020; Della Corte et al., 2020; Pessoa de Farias Rodrigues et al., 2020) have analysed the health of women affected by endometriosis, only few of these have used a qualitative approach. Indeed, most of them were focalized on clinical symptoms, diagnostic techniques and laboratory data (Denny & Khan, 2006; Young et al., 2015). Four studies analysed the lived experiences of women affected by endometriosis. For example, DiBenedetti et al. (2020) analysed the lived experiences of endometriosis related to fatigue, using a semi-structured interview. In this study, authors observed that most participants noted an impact from endometriosis-related fatigue on daily activities, social relationship, mood and emotion and working activities. In another study (Vennberg Karlsson et al., 2020), conducted on 12 persons with endometriosis, authors, through a semi-structured interview, analysed the experiences of health after dietary changes and observed that participants experienced an increase in well-being and a decrease in symptoms following their dietary and lifestyle changes. Differently, another study (Hållstam et al., 2018) analysed the women’s experience of painful endometriosis, observing that living with severe painful endometriosis signified a struggle for coherence.

However, although these studies were focalized to analyse the lived experiences of women with endometriosis, these studies analyzed only specific aspects of endometriosis such as fatigue (DiBenedetti et al., 2020), dietary changes (Vennberg Karlsson et al., 2020) or pain (Hållstam et al., 2018), while in our study, following the phenomenological methodology, we did not limit to analyse only one specific aspect, but the lived experiences in general. Knowing the lived experiences of women suffered by endometriosis, it is crucial to understand the physical and psychological implications that endometriosis could have on their lives, and consequently, give information to implement tailored intervention.

## Aim of study

The aim of the study was to understand the life experiences of women suffering from endometriosis.

## Method

### Design

For this study, a qualitative methodology was adopted: Cohen’s phenomenology (Cohen et al., 2000). This methodology, which differs from other phenomenological traditions, combines the characteristics of descriptive phenomenology (Husserliana) and interpretative phenomenology (Gadmeriana). While *descriptive phenomenology* tends to describe the general characteristics of the phenomenon, remaining free from external factors that can influence it, *interpretative phenomenology* describes, understands and interprets the experiences of individual participants (Tuohy et al., 2013). These experiences are obviously influenced by the social, political and cultural context and by the surrounding subjects (Flood, 2010).

Cohen’s phenomenology, which has been used in previous studies (Simeone, Platone et al., 2018; Simeone, Pucciarelli et al., 2018; Simeone et al., 2017; 2015), was chosen for its suitability for gaining a deeper understanding of the experiences and the meaning attributed to those experiences.

### Recruitment

Eligible participants were identified and enrolled by the healthcare providers of specialist centre for the treatment of endometriosis. Accordingly, the healthcare providers contacted the eligible patients, explained to them the aim of the study and offered information based on their interest in being enrolled.

The objectives and details of the study were explained by a researcher to each participant before acquiring informed consent. Participants were guaranteed the freedom to withdraw their consent to participate in the study at any time. Participants were assured of data confidentiality at all times. After the participants provided written consent, investigators assigned each patient a number, hiding their identities.

The inclusion criteria were: 1) at least 18 years of age; 2) able to speak and understand Italian; 3) participate in the study voluntarily; 4) diagnosed with endometriosis. This study did not receive any subsidies or sponsorships from third-party entities, companies or individuals.

### Data collection and analysis

Following the chosen methodology, the first step, for all researchers involved in the study, was “bracketing” (Cohen et al., 2000): putting aside all their preconceptions related to the phenomenon being examined. When phenomenological methods, using an interpretive approach, are employed, data analysis may be vulnerable to interference related to the researchers’ preconceived ideas. Bracketing generally refers to the interviewers’ identification of their personal experiences regarding the phenomenon under study. In this phase, cultural factors, hypotheses and intuitions that could alternate the analysis the study data are transcribed. Bracketing continues throughout the research process, and researchers perform it continuously (Fischer, 2009; Hamill, 2010). Putting aside all their preconceptions related to the phenomenon being examined, the researchers are required to write down all their beliefs, prejudices or hypotheses about the phenomenon that they intend to investigate. This passage, also called “critical reflection” (Cohen et al., 2000), reduces the possibility of introducing an investigator’s opinions into the data analysis, which should instead focus on the participants’ point of view. This imparts rigour to the data analysis. The interviews, which followed this step, were all conducted by two researchers. After making meeting arrangements with the interviewee, the interviewers went to the place chosen for the interview. The interviews were conducted in the participant’s natural environment of their homes. We interviewed the participants with the following single open question: “You suffer from endometriosis. Could you kindly describe your experience living with endometriosis?” Conducting the interview with a single open question allows participants to have full freedom of expression. During the interviews, the researchers carried a diary to the field wherein they took notes, such as information about the environment, the interview set-up, participants’ verbal and nonverbal cues and reflections.

Data saturation (Polit & Beck, 2014) was achieved after 25 interviews. Each interview was audio recorded; the average duration of each interview was approximately 50 minutes. To have full mastery of the selected methodology, the researchers conducted two interviews which were defined as test interviews, which were not included in the study. All the interviews were transcribed verbatim for data analysis. As required by Cohen’s methodology (2000), the individual text is understood in relation to all the texts and vice versa. The researcher begins with a vague and tentative notion of the meaning of the whole of the data and with the reflexive awareness that this notion is an anticipation of meaning. Once an understanding of the overall text is obtained, phrases in the text are underlined, and tentative theme names are written in the margin of the text. Data are examined line by line, and all important phrases are labelled with tentative theme names. This phase of the analysis requires that the investigators label themes and extract passages that have similar themes to be able to look at them together and alongside passages that have the same label but are separated from the rest of the text. Each researcher involved in this study first read and reread the interviews several times, together with the notes, to have an overall understanding of the patients’ experiences. Subsequently, each individual interview was reread and labelled with a provisional theme and then compared with other interviews. The individual researchers compared the various extrapolated themes. There was no discrepancy between researchers at this stage. The extrapolated themes were then confirmed by each participant during a second interview which took place about three weeks after the first interview. Here the researchers asked the participants to confirm the extrapolated themes. This ensured scientific rigour and final validity of the results. The data were analysed in Italian due to the nationality of the participants. An independent translator translated the data after it had been written. A second translation by a second independent translator was requested. The versions were compared to each other by the authors and no discrepancies were found.

## Ethical considerations

This study fully complies with the principles outlined in the Helsinki Declaration. Ethical approval was obtained prior to data collection by institutional review board XXX (Ethical approval n° 134/16). Participants interviewed in this study were selected from those involved in a larger ongoing Italian multi-site longitudinal study aimed at studying quality of life in patients with gynaecological. The participants signed an informed consent form after the study was explained to them before the interview. Participants were assured that their data did not contain identifying information and that they had the right to withdraw from the study at any time.

## Results

The study sample consisted of 25 women, with an average age of about 27 years, in a range that varied between 18 and 54 years. The endometriosis diagnosis had been made between 1 week and 30 years prior to participating in the study. The data analysis identified four main themes and two sub-themes.

The main themes were: nobody believed me, which included the sub-theme of the women not understanding their condition; worsening of the women’s lives, which included the sub-theme of painful lives; disastrous intimate lives with their partners; and uncertainty about being able to have children.

### Nobody believed me

A common theme among all women was that of delayed diagnosis of endometriosis. The definite diagnosis had often been made after years of investigations during which the symptoms of endometriosis were considered almost physiological. This delay impacted on their lives, affecting many aspects of their lives. During the period of time elapsed from the onset of symptoms until the final diagnosis, our participants underwent specialist medical visits and diagnostic tests. The difficulties to identify a cause related to their illness has certainly influenced their lived experiences. Not having a specific cause related to the symptoms involves a perception of non-healing, as well as the perception of the absence of specific treatment.

AZ01: “I had been going back and forth between examinations, visits and everything else for years … in the end I was at the point of giving up, I had almost convinced myself that I was just like this … then came the diagnosis… ” EV05 adds: “I had been told that I was unlucky, a painful menstrual cycle, with abdominal repercussions and other pains that were all probably related to that period … but then, eventually, there was the news about the disease”.

In addition, the absence of a specific diagnosis influences also their social life. The participants actually described a feeling of isolation, which was essentially due to a *misunderstanding of their situation*.

GT07: “I think we’ve all faced certain times, of course not all of us in the same way, but certain days for me were really extreme and yet, no one seemed to believe me. I felt isolated and angry … nobody believed me, but then the diagnosis arrived”. QL15: “..for a long time I felt like I was alone, nobody believed me… but I really felt so bad and it couldn’t have been physiological or just psychological… I was really sick, my body was saying so, but I was the only one who was listening to it”.

### Worsening of the women’s lives

Endometriosis and its symptoms, even prior to diagnosis, had a negative effect on all our participants, impacting their general quality of life adversely. Every aspect of their lives has been negatively affected. The participants found the important repercussions that their clinical situation had in the workplace, social and personal spheres. It seems that the disease influenced every daily activity; pain as described above and other symptoms affected performance at work, social relationships and also management of free time.

GT07: “…my performance at work has also decreased, if not in terms of quantity, certainly from a quality standpoint. I try to be super productive at the times that I feel well, or better … at the other times I try to avoid contact where possible. Before the diagnosis I sometimes skipped work inventing a thousand excuses”. IR09: “I started going out less even with my friends … and they didn’t always understand … they thought I was exaggerating or who knows what else”. CX03: “..some days I even neglect doing household chores and I feel guilty about that, but I really just can’t, I can’t do it”. DW04: “..even at night sometimes I can’t rest or relax, so the next day I’m exhausted and irritable.”

Specifically, the constant presence of pain upsets the lives of these women. Every decision is not in itself free from the influence of this sensation. The women have summarized how their lives have become ***painful lives***, or how pain has completely influenced their lives.

UF19: “..living constantly with pain stresses you, it mentally destroys you … you think about going out to have fun, but then your mind says to you, what if the pain starts? What if it gets worse? Will there be a bathroom? Can I still take this drug?” DW04: “those feelings are there all the time, every hour of the day and night … severe, excruciating, stabbing pain in the abdomen, in the private part, in the back … they destroy you and drain your strength”.

### Disastrous intimate lives with partners

Another major theme that emerged in common within our sample is the matter of their sex lives. The participants, probably also thanks to the methodology used, have clearly described how endometriosis has negatively influenced this part of their married life. The fear that the upheaval of their intimate life could negatively influence every aspect of the couple’s life constantly accompanies the participants. The importance attributed to intimate life, from a personal point of view and in relation to couple’s life, also led the participants to describe their strategies implemented to overcome the difficulties that arose in their life aspects.

MP11: “and then in the evening, when I used to be able to have moments of intimacy with my husband, well, I always end up refusing even though I actually want to be with him … it’s unpleasant and unfortunately it adds barriers, walls between us … it’s hard to get him to believe me. I feel guilty but I can’t help it”. QM14: “my sex life was deeply affected … the pleasure has almost completely disappeared … there is only the pain that causes you discomfort and increasingly makes you avoid any approach at all so that you won’t feel it again … different positions don’t change those unpleasant sensations … Sometimes we resort to other methods to feel pleasure”. HS08: “my sex life is almost over, I continually avoid my husband and sometimes fear betrayals or even divorce”. LQ10: “Intimacy is obviously an important part of being a couple and I’m not experiencing that, and that means I’m not satisfying my partner either … it’s not uncommon for us to reach pleasure without having traditional intercourse”.

### Uncertainty about being able to have children

The last theme that emerged is related to the uncertainty of reproduction. Having a baby is inherent in being a woman. Culturally, motherhood is a fundamental aspect of being a woman. It is seen as a natural event, as well as the good health of the unborn child. Our participants, mostly young women, expressed deep concerns and fears about the result that endometriosis could have on a potential completed pregnancy. Their concerns focus not only on the correct conduct of a pregnancy, but also on how the health of the foetus could be affected by the treatment of their chronic condition. Our sample, mostly young women, expressed deep concerns and fears about the result that endometriosis can have on a potential completed pregnancy.

ZA25: “and then I immediately wondered whether I could have children in the future. I was already feeling down, then that thought broke me completely”. YB23:“the fear of not being able to have children destroys me … it’s a thought that unfortunately impacts daily life and tends to isolate me because other people don’t understand, wouldn’t understand … a child means almost everything to a woman … the uncertainty about it scares me a lot, it stops me from making a lot of choices or decisions … ” XD21: ‘once there was went the diagnosis, or sentence, it triggered three thousand thoughts. When you’re having tests the mind wanders and you unfortunately end up searching for things on the internet that it would be better not to know … so my fertility was one of the first things on my mind when it was diagnosed. Will I be able to have children? Will drugs affect my pregnancy, my hypothetical baby … will the child be harmed? Will I be able to get pregnant? I know that endometriosis is linked to infertility, or almost … ‘

## Discussion

This study aimed to investigate the experiences of women suffering from endometriosis. The analysed data showed that all the participants faced long periods of uncertainty about the cause of their ailments, with diagnoses being made only after a long period of time. The time that passed between the onset of symptoms and receiving a definite diagnosis has had a significant impact on their lives not only physically but also psychologically and socially. During that time, the participants experienced social and personal isolation, even with their partners. Moreover, the doubts about their fertility that arose after the diagnosis likely had a negative effect on their mental health.

The theme of delay in the diagnosis of endometriosis, which we named “nobody believed me”, is a widely discussed topic in literature (Culley et al., 2013; Facchin et al., 2015; Moradi et al., 2014; Young et al., 2015). Though often seen as a challenge to be tackled positively (Moradi et al., 2014), in our study the delay in diagnosis was instead a critical factor in worsening the participants’ quality of life (Nnoaham et al., 2011). Our sample has not overtly expressed any disappointment in the health workers (Grogan et al., 2018)—neither for the delay in diagnosis nor for the feeling of being understood. The cultural values of our sample (Simeone et al., 2016) may play a significant part in this. Initially, common symptoms of endometriosis are often considered to be normal in women (Cox et al., 2003; Culley et al., 2013; Elaine Denny, 2004; Laws, 1990), especially for teenagers (Manderson et al., 2008). In our study, we saw how the uncertainty of diagnosis could have an impact on a person’s life. Our participants feel worried precisely because they are unable to receive treatment. Uncertainty is cause for concern and this could also have major repercussions on the quality of life. Furthermore, our results highlight how important it is to provide psychological support to women who are diagnosed with endometriosis. Indeed, as described by some participants, they felt angry, isolated, the only ones who could understand the situation. Psychological support could make this situation better and not create emotional distress for women. However, within our sample, this delay seems to have significantly contributed to underlining the participants’ feeling of not being understood. Since our participants did not have a definite diagnosis, they felt that people around them did not believe that they were unwell, and that only increased their sense of isolation. Decisions relating to their social lives were significantly influenced by the symptoms of endometriosis (E. Denny, 2004; Gilmour et al., 2008).

The worsening of their lives was the second common theme for all our participants. Almost as a consequence of what has been expressed above, our participants clearly felt and reported how endometriosis, and its prior symptoms, have profoundly influenced their lives in a strongly negative way. Endometriosis has a big impact on people’s lives, as evidenced by our results, especially in the workplace. Women, especially those who suffer from endometriosis, must live with all the problems related to this situation and at the same time have to be “super” (as described by a participant) as wives, mother, and workers. We see that our participants highlight how destructive endometriosis is for their physical and emotional well-being. A constant pain that affects any daily life activity. Existing studies in literature show that endometriosis is linked to a worsened quality of life for the women suffering from it, showing higher rates of depression, anxiety and emotional stress compared to the normal population (Culley et al., 2013). The symptoms of endometriosis, as described above, significantly impacted every aspect of the participants’ lives (E. Denny, 2004; Fauconnier et al., 2013; Huntington & Gilmour, 2005), almost as though pain was controlling their lives (Denny, 2009; Huntington & Gilmour, 2005). Their stories have identified, as reported in the literature (Young et al., 2015), that pain is the main symptom responsible for changes in their social and working lives (Gilmour et al., 2008).

The influence that endometriosis had on the participants’ intimate relationships was clearly identified and described by the participants. Endometriosis also has an impact on couple life. Endometriosis represents a barrier for women especially in aspects related to intimacy, to the point of being worried that this could lead to a divorce. This is addressed in literature and the results of existing studies are similar to the ones in our sample: pain during sexual intercourse, which is considered important for life as a couple, leads to tension and misunderstanding (Culley et al., 2013; Denny & Mann, 2007; Fauconnier et al., 2013; De Graaff et al., 2013). However, some of our participants described strategies that they had implemented to facilitate or complete their sexual relationship (Young et al., 2015). Some women also described non-penetrative sex. In the literature, only one study (Denny & Mann, 2007) investigated the coping strategies used by women with endometriosis to explore non-penetrative or alternative sexual activity. The methodology used has likely allowed for such intimate and detailed descriptions. These findings highlighted also the importance to implement a sexual therapy after diagnosis of endometriosis. When sexual difficulties are particularly worrying and distressing, sexual therapy can be very useful, and could represent a rather pleasant experience for the couple. Sexual therapy is a particular therapeutic technique specifically designed to help people facing sexual difficulties, created with the aim of improving their physical intimacy. For example, sexual therapist could suggest relaxation and communication techniques, which could facilitate better physical or emotional relationships and open and honest communication between women and their partners. However, further research is needed to confirm the potential beneficial effects of sexual therapy on endometriosis patients and their love life.

The last theme that emerged was related to potential infertility. After receiving the diagnosis of endometriosis and having it explained to them, the women’s minds seemed to immediately jump to the issue of motherhood. The participants in the study clearly described how the possibility of not being able to have children affected their perceived quality of life (Jones et al., 2004). In addition to infertility linked to the disease, some of our participants also wondered about the impact drugs could have on a hypothetical pregnancy. Our participants also described how motherhood was an integral part of being a woman (Cox et al., 2003). However, for something that is considered so fundamental, it has received very little attention, especially considering that about half of the women with endometriosis can be infertile (Culley et al., 2013).

### Strengths and limitations

This study has several implications. The findings of our study may stimulate further qualitative and quantitative studies of women affected by endometriosis. The number of women who participated in the survey and the detail of their responses are the main strengths of this paper. Our findings suggest that support that husband of women affected by endometriosis could give, developing interventions which may be best promoted by family-centred care programs. A family-centred care program, where both husband and women with endometriosis could be involved, could be a specific approach to improve women’s anxiety, depression and fear.

However, this study has also several limitations. The monocentricity of the participants’ recruitment is the study’s main limitation. As with all qualitative studies, our findings cannot be generalized and can be used in other countries only with caution. The study was conducted only in one Italian region (Campania), and slight cultural differences between Italian regions and other countries could exist.

## Conclusions

Endometriosis is a disease with late diagnosis, which cannot be cured and is highly disabling not only for the female population affected by this disease. The costs associated with its treatment are significant, as are the considerable repercussions for the perceived quality of personal, couple, family and social life. Our results show that the delay in the diagnosis of endometriosis has had important consequences on perceived quality of life of the affected women such as on social, personal and working life. These aspects are influenced in the present and also in the future expectations that women could have. Furthermore, the fear that this situation could affect life with one’s partner and the uncertainty about the possibility of having children are two other frequent themes in the women who participated in our study. This could have psychological repercussions and therefore it would be advisable to provide psychological support. Understanding how this disease affects the lives of women who suffer from it can help create targeted intervention programmes.

## Strengths and limitations

- Cohen’s phenomenological analysis allows researchers to analyse complex data that provide new knowledge on the understanding of the experiences of women suffering from endometriosis.
- This study investigated the psychosocial aspectof endometriosis that is still little investigated by international researchers.

The monocentricity of the sample and the qualitative approach do not allow generalisation.
